# Arterial pulse waveform as an n-soliton evolution of the left ventricular pressure pulse

**DOI:** 10.1186/cc13312

**Published:** 2014-03-17

**Authors:** B Feix, A Ercole

**Affiliations:** 1 Division of Anaesthesia, University of Cambridge, UK

## Introduction

The time profile of the arterial pulse is known to have features such as the dicrotic notch and subsidiary peaks. Such features may provide useful information about the vascular system and are traditionally explained in terms of aortic valve closure or multiple reflections from impedance mismatches within the arterial system. However, experimental evidence of such reflections has been elusive. It has been proposed that arterial dynamics may obey a nonlinear equation [1]. This model predicts the existence of multipeaked solitons which can travel long distances without dissipation. We demonstrate that within the soliton model it is not necessary to model valve closure or wave reflection: single or multiple notches arise *de novo *even from featureless theoretical LV pressure pulse profiles. We show that a number of clinically relevant features of the invasive blood pressure are reproduced by the soliton model and examine the role of LV pulse energy on pulse wave shape and progression.

## Methods

A model for the arterial pressure is given by solutions to a KdV equation with constants depending on the properties of the artery [1]. This can be solved with the initial condition of a parabolic left ventricular pressure pulse.

## Results

**Figure 1:**
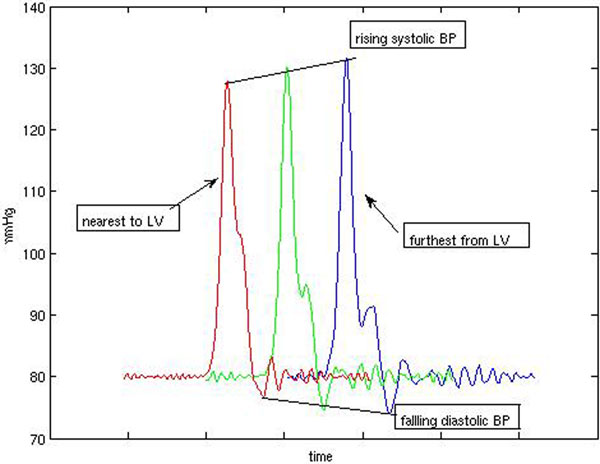


The evolution of the arterial pulse along the arterial tree is shown in Figure 1. We also predict arterial pulses for increasing left ventricular ejection energies.

## Conclusion

Our simple model explains many features of the arterial pulse observed in clinical practice such as the development of the dicrotic notch, the change in shape along the arterial tree and the steepening and acceleration with hypertension. Some phenomena that have traditionally been attributed to arterial wave reflections or resonance of the invasive arterial pressure measurement can instead be explained by intrinsic properties of the arterial pulse.
